# Chronic Household Air Pollution Exposure Is Associated with Impaired Alveolar Macrophage Function in Malawian Non-Smokers

**DOI:** 10.1371/journal.pone.0138762

**Published:** 2015-09-25

**Authors:** Jamie Rylance, Chikondi Chimpini, Sean Semple, David G. Russell, Malcolm J. Jackson, Robert S. Heyderman, Stephen B. Gordon

**Affiliations:** 1 Liverpool School of Tropical Medicine, Liverpool, United Kingdom; 2 Dept of Respiratory Medicine, Clinical Sciences Centre, University Hospital Aintree, Liverpool, United Kingdom; 3 Malawi-Liverpool-Wellcome Clinical Research Programme, Blantyre, Malawi; 4 Scottish Centre for Indoor Air, Division of Applied Health Sciences, University of Aberdeen, United Kingdom; 5 Microbiology and Immunology, College of Veterinary Medicine, Cornell University, Ithaca, New York, United States of America; 6 Institute of Ageing and Chronic Disease, University of Liverpool, Liverpool, United Kingdom; The Ohio State University, UNITED STATES

## Abstract

**Background:**

Household air pollution in low income countries is an important cause of mortality from respiratory infection. We hypothesised that chronic smoke exposure is detrimental to alveolar macrophage function, causing failure of innate immunity. We report the relationship between macrophage function and prior smoke exposure in healthy Malawians.

**Methods:**

Healthy subjects exposed daily to cooking smoke at home volunteered for bronchoalveolar lavage. Alveolar macrophage particulate content was measured as a known correlate of smoke exposure. Phagocytosis and intraphagosomal function (oxidative burst and proteolysis) were measured by a flow cytometric assay. Cytokine responses in macrophages were compared following re-exposure in vitro to wood smoke, before and after glutathione depletion.

**Results:**

Volunteers had a range of alveolar macrophage particulate loading. The macrophage capacity for phagosomal oxidative burst was negatively associated with alveolar macrophage particulate content (n = 29, r^2^ = 0.16, p = 0.033), but phagocytosis per se and proteolytic function were unaffected. High particulate content was associated with lower baseline CXCL8 release (ratio 0.51, CI 0.29–0.89) and lower final concentrations on re-exposure to smoke in vitro (ratio 0.58, CI 0.34–0.97). Glutathione depletion augmented CXCL8 responses by 1.49x (CI 1.02–2.17) compared with wood smoke alone. This response was specific to smoke as macrophages response to LPS were not modulated by glutathione.

**Conclusion:**

Chronic smoke exposure is associated with reduced human macrophage oxidative burst, and dampened inflammatory cytokine responses. These are critical processes in lung defence against infection and likely to underpin the relationship between air pollution and pneumonia.

## Introduction

Environmental smoke exposure is strongly associated with all-cause mortality, even at low concentrations [1]. Household air pollution (HAP) is the greatest source of human particulate exposure, and 3^rd^ most important risk factor for ill-health worldwide [2]. HAP associated respiratory infection results in 900000 excess deaths per year in children under 5 years [3], presumably due to adverse effects on airway immunity. These mechanisms are not understood, but the alveolar macrophage is central to both host defence and particulate handling.

Alveolar macrophages (AM) orchestrate appropriate responses to infective insults. AMs internalise bacteria, such as *Streptococcus pneumoniae*, activating NADPH oxidase which both kills bacteria and has a role in promoting inflammatory cytokine release [4, 5]. After successful containment, these responses are limited to prevent collateral lung damage [6]. However, inhaled particulate material taken up by AM has the capacity to induce excessive inflammatory signalling [7]. HAP wood smoke particles induce free radicals generation which augment macrophage NF-κB activation [8], reduce intracellular glutathione [9], and potentiate TNFα, IL-6 and CXCL8 release [10]. Adsorbed lipopolysaccharide has an additional pro-inflammatory effect [11].

In animal models, pneumonia mortality is associated with inadequate anti-inflammatory responses and reduced pulmonary macrophage apoptosis [12, 13]. Combined infective and particulate challenges were shown to exaggerate murine pulmonary inflammatory responses, and oxidative stress: phagocytosis of *S*. *pneumoniae* was impaired, and survival from pneumococcal pneumonia reduced [14]. In an alternative (high mortality) murine model, particle exposure improved survival, probably due to early neutrophil recruitment [15].

Acute particulate exposures in humans generate pro-inflammatory responses. Firefighters acutely exposed to wood smoke had increased systemic CXCL8 and neutrophilia [16]. Experimental human wood smoke exposure showed increased exhaled nitric oxide and malondialdehyde suggesting pulmonary inflammation and oxidative stress [17].

Chronic exposures appear different. Rats after 70 days of wood smoke exposure have minimal changes in BAL concentrations of cytokines, and lower levels IL-1β than controls [18]. There are few human data on pulmonary responses to chronic ambient particulate exposure, although cigarette smoking causes an hypo-responsive state in the *ex vivo* human alveolar macrophage, with blunted pro-inflammatory cytokine responses [19].

Previously we have reported preliminary data from a cohort of Malawians, suggesting reduced oxidative burst in alveolar macrophages with high particulate content [20]. We wished to perform a definitive study to extend these findings to other phagosomal functions, and to establish potential cellular explanations. Here, we hypothesise that chronic exposures reduce human macrophage phagosomal capacity to internalise and kill potential pathogens. Our hypothetical mechanism was that chronic exposure to smoke results in antioxidant buffering, altering redox balance in the macrophages, thus disrupting inflammatory responses important for defence against infection. We investigated macrophage function following natural ambient and household air pollution exposure in a biomass fuel burning adult population in Malawi.

## Methods

Ethical approval was granted by the Research and Ethics Committees of the College of Medicine, University of Malawi (P.03/10/916) and Liverpool School of Tropical Medicine (09.69). All participants gave informed written consent for their participation. Healthy non-smoking and HIV negative participants were recruited for bronchoscopy in Blantyre, Malawi. Bronchoalveolar lavage (BAL) to obtain alveolar macrophages (AM) was performed as previously described [20]. All assays were done at the site of sample collection (Malawi) excepting cytokine measurement.

### Cell culture

BAL was filtered through gauze and centrifuged. The pellet was re-suspended in RPMI1640 with 10% FBS and 2mM L-glutamine (culture medium) with penicillin, streptomycin and neomycin (Sigma-Aldrich, UK). At five hours culture medium was exchanged for that without antibiotics. AM were used on day 1 of culture after 4 hours of incubation at 37°C, 5% CO_2_.

Peripheral blood mononuclear cells (PBMC) were isolated from heparinised whole blood by Lymphoprep gradient separation (Axis-Shield, UK) and sequentially centrifuged according to manufacturer’s instructions. Cells were seeded at 7.5x10^5^/cm^2^. Adherent PBMC were incubated at 37°C 5% CO_2_ in antibiotic-free culture medium for 5 days before use, with fresh medium introduced every 2 days.

### Particulate Matter

Dried mopane firewood from Malawi (typically used for cooking) was transported to the UK and burned using a 3-stone open fire method in a contained room. Respirable sized particles (<4micron) were collected by a Higgins-Dewell cyclone at 2.2l/min attached to an Apex pump (Casella CEL, UK), onto polycarbonate isopore filters (Millipore, USA). Particulates were removed from filters by vortexing and sonication in methanol, dried under nitrogen gas and stored at -80°C.

**Fig 1:**
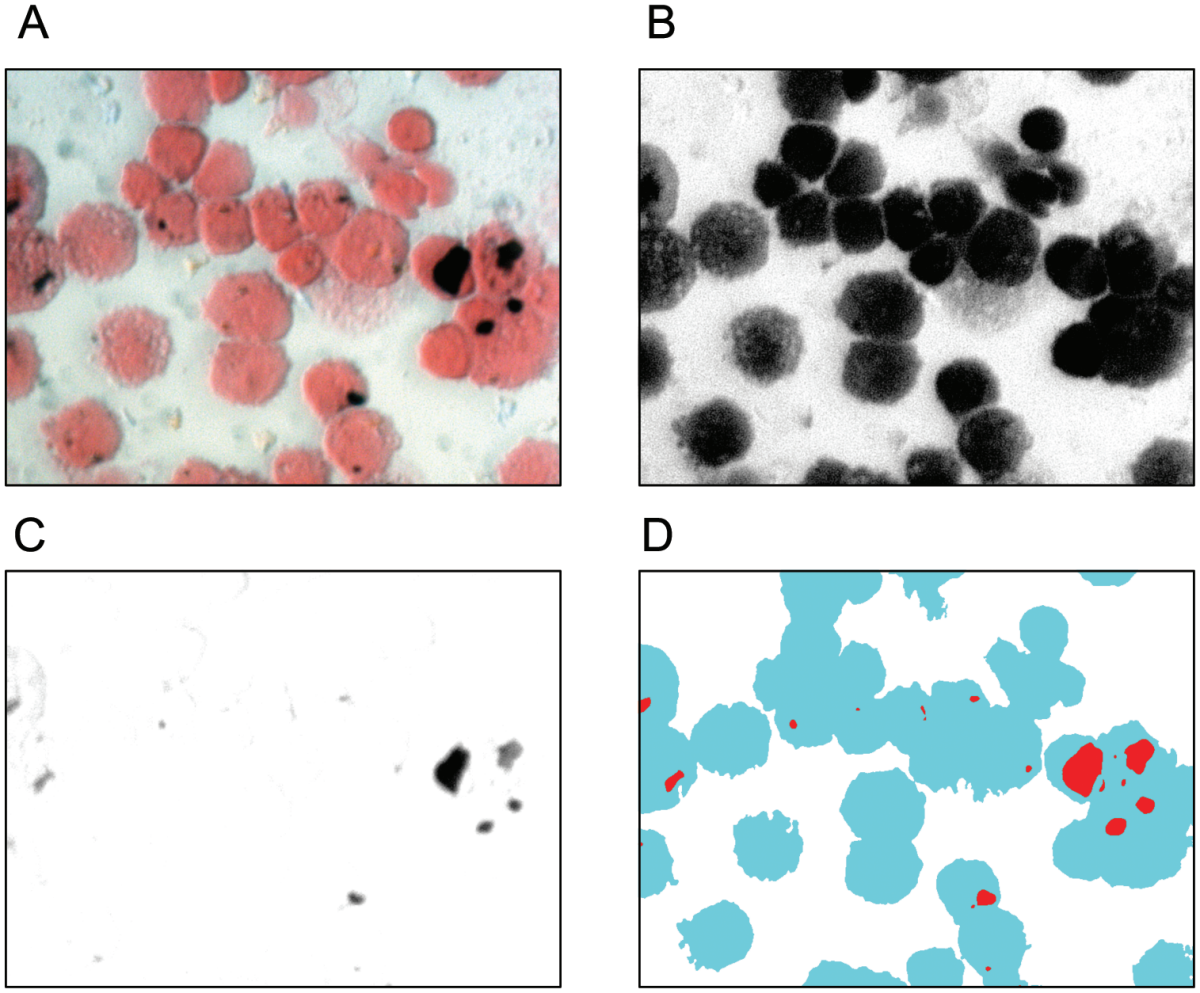
Particulate burden is measured by the percentage of macrophage cytoplasm taken up with particulate matter as calculated by digital image analysis of light microscopy images. ImageSXM software analyses cytospin images treated with Fields B stain (A), and identifies both cytoplasmic (B) and particulate areas (C). The proportion of particulate to cytoplasm in the output image (D) is used as a measure of recent particulate exposure. Fifty 40x fields are used, and the overall mean particulate burden used as the summary statistic. Panels B and C are generated internally within the software, and are shown here for illustrative purposes only.

We previously reported that the particulate content of AM correlates with reported exposure to household air pollution [21]. Particulate burden has been used as a biomarker of exposure [22], and was quantified by digital image analysis as previously described [20]. Briefly, cytospin preparations (Thermo Shandon) were imaged at 40x by light microscopy. Fifty fields from each experiment were analysed using freely available software (Image SXM, www.ImageSXM.org.uk)—see Fig 1 for diagrammatic details).

### Phagosomal assays

We used our previously validated flow cytometric assay to determine intraphagosomal oxidative burst and proteolytic functions [23]: silica beads linked to two fluorogenic probes (a reporter and a calibrator) are coupled to IgG to facilitate F_c_ receptor mediated uptake. Adherent alveolar macrophages were incubated with beads for 60mins and evaluated by flow cytometry (CyAn ADP, Beckman Coulter). Phagocytosis was defined as the proportion of cells associated with beads by flow cytometry. An example of analysis using FlowJo (Tree Star, Oregon, USA) is shown in supporting information S1 Fig Oxidative burst activity within the phagosome increases the fluorescence of bead-bound “reporter” 2',7'-dichlorodihydrofluoresceindiacetate succinimidyl ester (DCFHDA-SE, Invitrogen, UK). Similarly, proteolytic activity reduces quenching of bead-bound DQ-BSA (Molecular Probes), thereby increasing its fluorescence. For each assay, reporter fluorescence was compared to the stably fluorescent calibrator fluorochrome (Alexa 633, Invitrogen, UK) in order to control for variation in acquisition parameters. Both measures are reported as “activity index”, or the increase in ratio of reporter: calibrator fluorescence at 60 minutes (oxidative burst) and 180 minutes (proteolysis) compared with baseline.

### Stimulation experiments

Particulates from wood smoke (WS) were re-suspended in culture medium at 50μg/ml. This dose induces similar particulate burdens in alveolar macrophages to those seen in heavy chronic exposures [20]. Lipopolysaccharide from *E*. *coli* 0111:B4 (LPS) was used as a stimulus at a final concentration of 100ng/ml. WS and LPS stimulation of adherent alveolar macrophages was performed after 24 hours of *in vitro* culture after bronchoscopy, and samples stored at -80°C pending analysis. Cytokines were pre-selected based on their known effects on macrophages, and putative involvement in redox signaling, and assayed from supernatant using the Luminex platform (R&D systems, UK).

To induce oxidative stress in cytokine stimulation experiments, cells were pre-treated for 18hours with 0.2mM buthionine sulfoximine (BSO) causing glutathione depletion. Additional supplementation with 2mM N-acetylcysteine (NAC) provided antioxidant depletion/repletion conditions. BSO related cell toxicity was determined from lactate dehydrogenase concentrations in culture supernatants (Tox7 kit, Sigma).

### Redox assessment

Macrophages were lysed in 5% sulfasalicylic acid. Oxidised glutathione (GSSG) and total glutathione were measured in triplicate by a recycling enzymatic assay. Briefly, reduced glutathione is oxidised by 5,5’-dithiobis-2-nitrobenzoic acid (DTNB) to give a yellow product (TNB). Oxidised glutathione is recycled through the assay by NADPH dependent glutathione reductase. Total glutathione is determined from the linear reaction kinetic measured by absorbance at 412nm against known standards. Oxidised glutathione is identically measured, after pre-treatment with 2-vinylpyridine to remove reduced glutathione. Reduced glutathione (GSH) was calculated from [total glutathione = 2GSH + GSSG]. All reagents were obtained from Sigma, UK.

### LDH assay for cell toxicity

Toxicity resulting from BSO treatment was measured by lactate dehydrogenase release into culture supernatants using a kit (Tox7, Sigma) according to manufacturer’s instructions. NADH formed in the catalytic reduction of NAD+ reacts with iodonitrotetrazolium producing a blue formazan product which can be quantified by absorption at 492nm. Separately, alveolar macrophages treated with 1% Triton-X for 5 minutes at 21°C were centrifuged at 10000g for 5 minutes, and the supernatant used as a positive control.

### Data Analysis

Experiments were performed sequentially due to cell numbers. For each assay, consecutive participants were used to reduce the risk of bias. Due to the ubiquity of particulate exposure in the study population, control groups were not available. Results are therefore reported based of relative exposures within the spectrum of that measured.

Human AM production of cytokines, phagocytosis and phagosomal oxidative function was compared in subjects with variable exposure to household air pollution, measured by AM particulate burden. Cytokine data and particulate densities were normalized by logarithmic transformation, using x’ = log(x+0.05), due to zero values and to enable parametric testing. Groups were compared with paired t-test or ANOVA. Effects on cytokine release were assessed by Sidak’s multiple comparison testing. Analysis used Stata v12 (StatCorp, TX) and GraphPad Prism v6 (GraphPad, CA), with significance at p<0.05 unless stated.

## Results

### Participants

We recruited 128 non-smoking participants with median age 28.4 (IQR 23.6–34.9). 57 (44%) were female. Median bronchoalveolar lavage return was 65% (130ml, IQR 112–140), containing 96.3% macrophages (IQR 93.7–98.1) with 97% viability (IQR 95–99). Tobacco smoking by a household contact was reported in 23 (18%), but smoking in Malawi is outdoors and typically fewer than five cigarettes per day. Baseline details of participants are given in Table 1.

**Table 1:** Summary details of the participants’ demographics and BAL findings.

	n (%)
Age, mean years (SD)	30 (7.7)
Sex, female (%)	57 (44%)
Body Mass Index, mean kg/m^2^ (SD)	22.5 (3.8)
African ethnicity, n (%)	128 (100%)
Passive cigarette smoke exposure, n (%)	23 (18%)
Cooking (main source)	
Open wood fire, n (%)	43 (33.6%)
Charcoal stove, n (%)	76 (59.4%)
Electricity, n (%)	9 (7.0%)
Cooking location (predominant)	
Inside the house, n (%)	77 (60.6%)
Outside, n (%)	50 (49.4%)
Lighting (main source)	
Paraffin, n (%)	45 (35.4%)
Candle, n (%)	47 (37.0%)
Electricity, n (%)	31 (24.4%)
Flaming torch, n (%)	4 (3.2%)
Incomplete data	1
FEV_1_, mean % predicted (SD)	94 (13.5)
FVC, mean % predicted (SD)	98 (12.6)
FEV_1_/FVC, % (SD)	82 (6.7)
BAL return*, median ml (IQR)	130 (112–140)
Cell viability, median % (IQR)	97 (95–99)
Percent of BAL macrophages, median (IQR)	96.3 (93.7–98.1)
Macrophage particulate load, median % of cytoplasm (IQR)	0.4 (0.2–0.9), range 0–8

SD standard deviation; IQR interquartile range;

* 200ml instilled.

### Particulate loading in macrophages from subjects exposed to HAP

121 (94.5%) participants were routinely exposed to biomass derived household air pollution: 119 (93%) used biomass fuel for cooking of whom 77 (61%) cooked only indoors; 94 (75%) used candle or paraffin as the main source of lighting, the remainder used electricity or battery powered lights. Median macrophage carbon load was 0.4% of total cytoplasmic area (range 0–8.0%, IQR 0.2–0.9%).

### Intraphagosomal function

**Fig 2:**
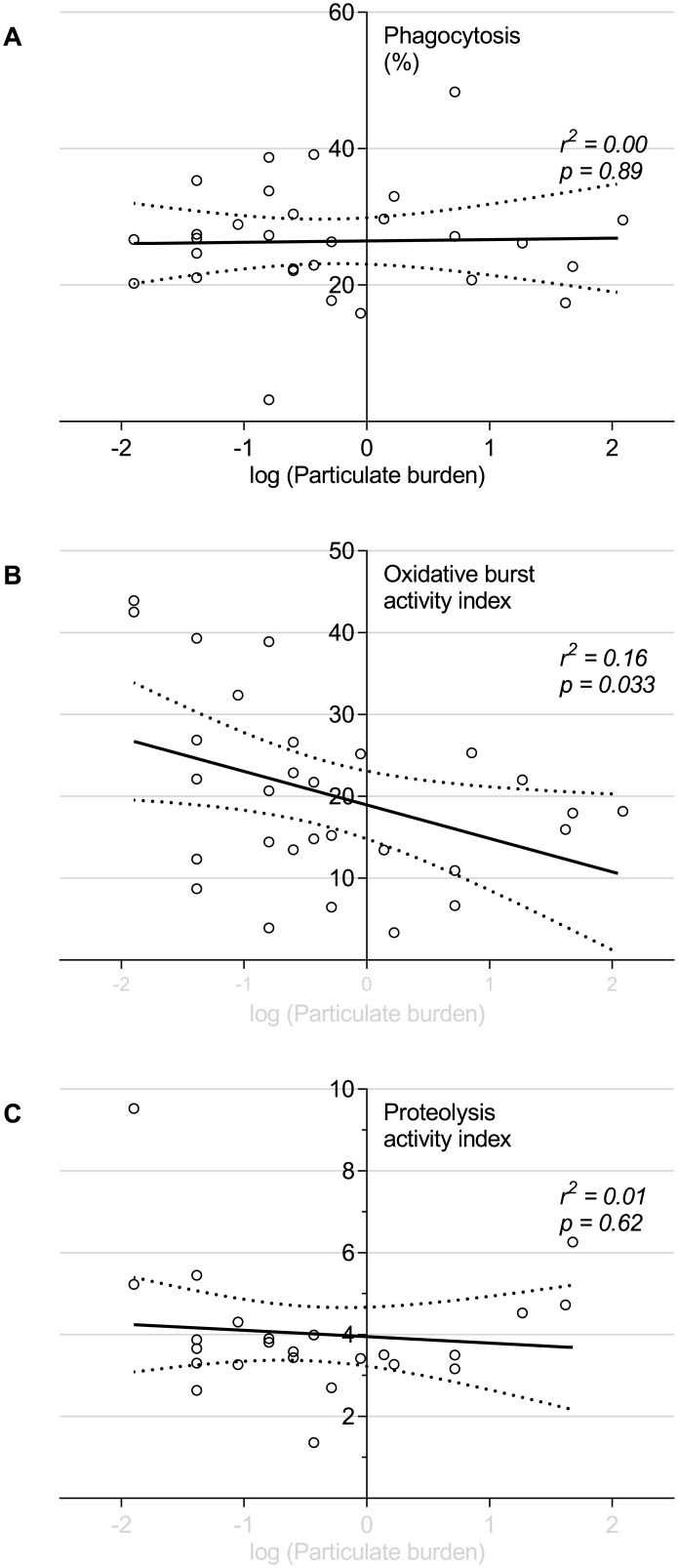
Alveolar macrophages naturally exposed to higher levels of particulate have reduced capacity for intraphagosomal oxidative burst, but an unaffected capacity for phagocytosis and proteolysis. Unselected participants naturally exposed to inhaled particulates underwent bronchoscopy. The burden particulate within the alveolar macrophage was used as a measure of overall exposure. The capacity of alveolar macrophages to phagocytose, produce oxidative burst and proteolytic responses was tested on the same day by co-incubation with fluorophore labelled silica beads. (A) phagocytosis as indicated by the percentage of macrophages associated with fluorophore labelled beads by flow cytometry. (B) Oxidative burst and (C) Proteolysis were measured using twin labelled beads with calibrator fluor and reporter fluor (DCFH-DA and DQ-bovine serum albumin conjugate respectively), reported as “activity index” (see Methods). Each dot represents an individual (n = 29). Solid and dotted lines show the linear regression model with 95% confidence intervals. The x-axis describes the particulate burden (% of cytoplasm taken up by particulate). This has been log transformed by x’ = log(x+0.05), as described in the methods in order to normalise the data.

Phagocytosis, proteolysis and oxidative burst were measured in 29 BAL samples. There was a significant negative correlation, between macrophage particulate content and macrophage intraphagosomal oxidative burst (see Fig 2, r^2^ = 0.16, p = 0.033). There were wide differences between individuals in propensity to generate phagosomal reactive oxygen species (ROS). Neither phagocytosis *per se* nor proteolytic function were associated with macrophage particulate loading (r^2^ = 0.00, p = 0.90; r^2^ = 0.01, p = 0.25 respectively).

### Cytokine response and particulate burden

**Fig 3:**
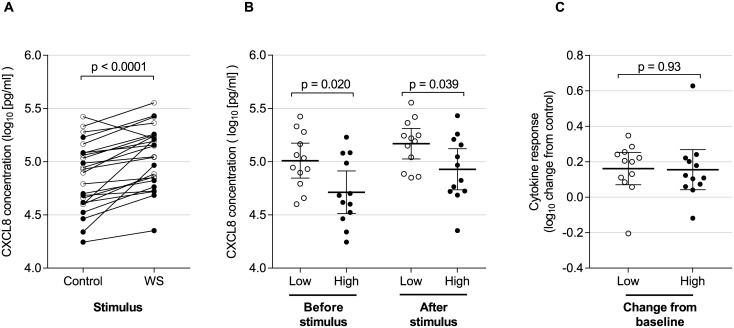
CXCL8 release is decreased in particulate laden macrophages. Prior exposure to particulates was measured by HAM particulate burden. Individuals were designated “low” and “high” exposures according to whether their HAM particulate burden was below or above the median value for this group (n = 24). Adherent HAM on day 1 after bronchoscopy were incubated with media only or with additional wood smoke suspension for 6 hours. CXCL8 concentrations in the cell culture supernatant following challenge are shown logarithmically transformed (log_10_). For all panels, open circles represent individuals with “low” macrophage particulate burden. Filled circles are those with “high” baseline particulate burden. (A) shows concentrations of CXCL8 in media for control and wood smoke stimulated wells for the entire group. There is a significant increase in concentration in stimulated cells (paired t-test, p<0.0001). (B) shows CXCL8 concentrations from untreated (control) and wood smoke treated wells. Here, results are grouped according to baseline particulate burden (low and high as described above). Bars show the mean and 95% confidence intervals. There is a significantly higher CXCL8 concentration from cells with low baseline particulate burden in both control and wood smoke treated wells (two-sided t-test, p = 0.020 and p = 0.039 respectively). (C) represents the increase in CXCL8 (log_10_ change in wood smoke treated wells compared with control). Bars show the mean and 95% confidence intervals. There is no difference in the magnitude of cytokine response between cells with low and high baseline particulate burden (two sided t-test, p = 0.93).

AM from 24 consecutive Malawian participants were stimulated *ex vivo* with wood smoke extract or media control. Culture supernatant CXCL8 concentrations rose at 6 hours (1.54 fold increase above control [95%CI 1.30–1.82], p<0.0001), see Fig 3A. We compared baseline CXCL8 production between low and high particulate groups as defined by the median macrophage particulate content. AM with high particulate loading had lower CXCL8 release compared with low particulate under unstimulated culture conditions (ratio of means 0.51 (95%CI 0.29–0.89, p = 0.02), see Fig 3B. After stimulation with wood smoke, this difference persisted: the ratio of mean CXCL8 in high compared with low particulate groups was 0.58 (95%CI 0.34–0.97). There was no relationship, however, between high particulate loading and the magnitude of CXCL8 responses (ratio of mean high vs low of 0.99 (95%CI 0.72–1.35, p = 0.93), see Fig 3C.

### Cytokine response and redox balance

**Fig 4:**
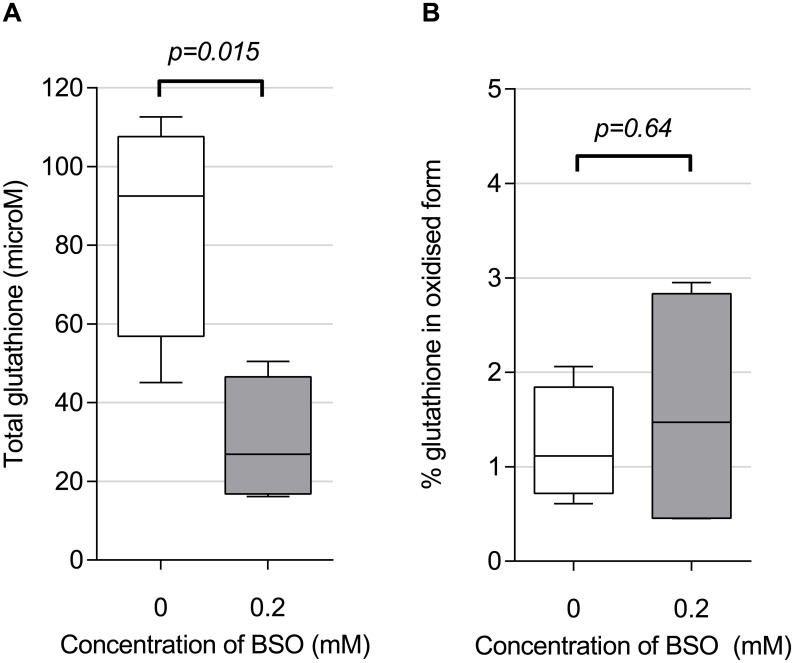
BSO treatment of HAM reduced total intracellular glutathione concentrations but did not alter overall proportion of glutathione in the oxidised state. *Ex vivo* adherent macrophages (3x10^6^ per well) were cultured for 18 hours in medium only (control), or with addition of 0.2mM BSO. Concentrations of total and oxidised glutathione were measured using an enzymatic recycling assay (see Methods). (A) Total glutathione (a measure of buffering capacity against oxidative stress) is reduced by BSO treatment compared with control. (B) Oxidised glutathione, as conventionally expressed as a percentage of total (a measure of oxidative stress), was not significantly altered by BSO treatment. n = 4 in each group. Boxes indicate 25^th^ to 75^th^ centile with median, and whiskers at 95% CI. Two sided t-test used for comparison.

Having described the relation of particulate load to both cytokine production and phagocytic function, we then determined the extent to which AM cytokine responses to particulates (stimulated *ex vivo*) were dependent on intracellular redox balance. Preliminary experiments (Fig 4A) showed that buthionine sulfoximine (BSO) 0.2mM decreased total glutathione (total glutathione) in HAM to 35% of its baseline (from 85.7μmol [SD 14.3] to 30.1μmol [SD 8.0], p = 0.015). BSO treated AMs therefore had reduced antioxidant buffering capacity (total glutathione). However, the ratio of oxidized to total glutathione (GSSG:total glutathione) expressed as a percentage was unaltered: control and treated mean values were 1.2% (SD 0.30) and 1.6% (SD 0.66) respectively (Fig 4B). This ratio is a frequently used measure of redox signaling [24], and suggests that the macrophage redox potential was not significantly affected, at least under resting culture conditions. There was no evidence for BSO induced toxicity (supporting information S2 Fig).

**Fig 5:**
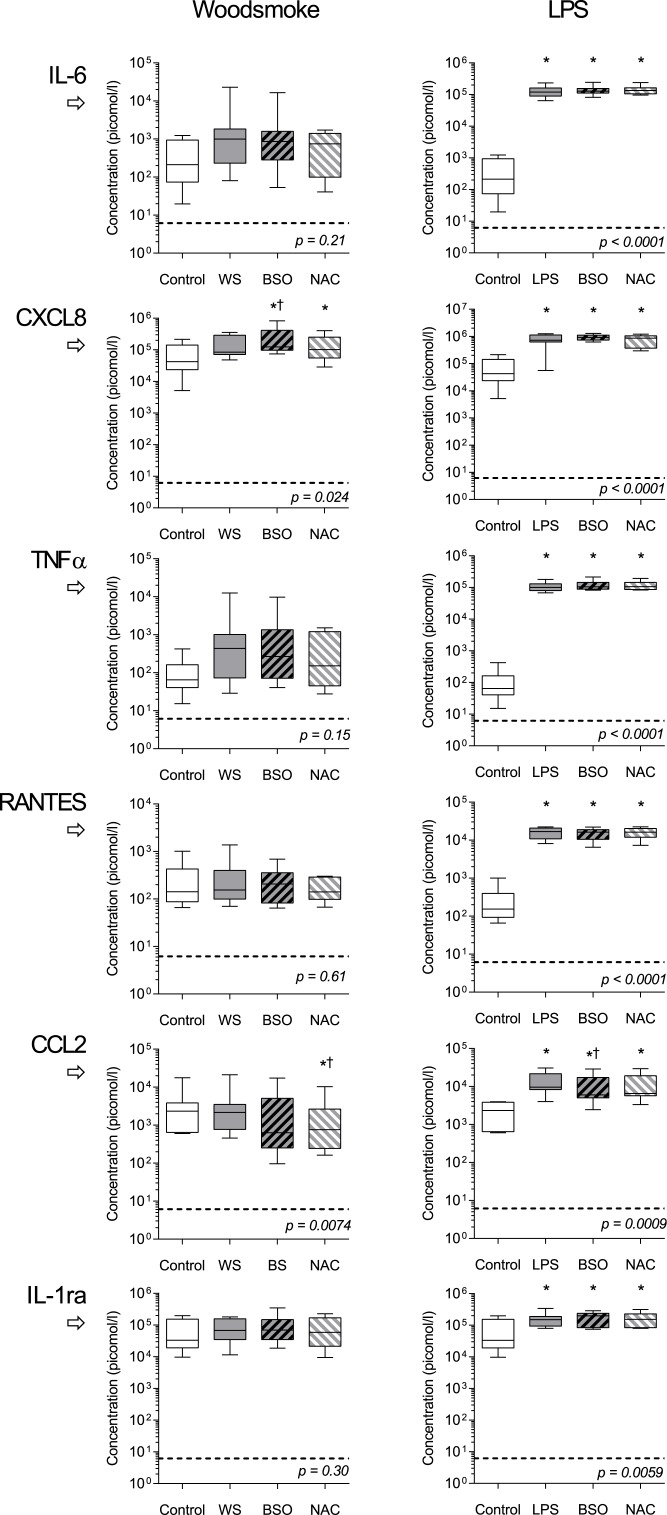
Cytokine responses to wood smoke particulates and LPS: the effect of intracellular redox balance. *Ex-vivo* adherent alveolar macrophages were pre-incubated with media alone (control), 0.2mM BSO (glutathione depletion), or BSO plus 2mM NAC (antioxidant depletion-repletion) for 18 hours, followed by stimulation with either media alone (control), wood smoke (left panels, WS) or lipopolysaccharide (right panels, LPS). After WS stimulus, low level changes were seen in CXCL8 and CCL2 release, while other cytokines were no significantly different. Maximal responses were seen with LPS, with no consistent effect of redox manipulation. Boxes show mean cytokine concentration in cell culture supernatants with 25^th^ to 75^th^ centile, and whiskers at 95% CI. n = 8 for each group. p values represent repeated measures ANOVA on log-transformed data. * denotes difference from control. † denotes difference from stimulated condition without pre-treatment. Significance testing by Sidak’s multiple comparisons test (p<0.05 significance level). Horizontal broken line represents limit of detection for each cytokine.

Cytokine release from BSO treated AM was measured following *ex vivo* incubation with WS particles and LPS (Fig 5). CXCL8 production was augmented by BSO pre-treatment (mean fold increase of 1.49 [95%CI 1.02–2.17]). NAC offset this increase. CCL2 release was also altered by redox balance (p = 0.0074 by ANOVA), and was significantly reduced in response to wood smoke in cells pretreated with BSO/NAC (mean fold difference 0.40 [CI 0.24–0.66]. Other inflammatory mediators demonstrated no statistical difference after redox manipulation. All measured cytokines were increased following lipopolysaccharide treatment (p<0.05 by repeated measures).

## Discussion

This study has shown that human AM obtained from participants most exposed to HAP have altered macrophage function: macrophage cytokine responses to wood smoke particles are reduced, and phagosomal oxidative burst responses are diminished. Further, we have shown that experimental alteration of human AM redox balance augments the prominent cytokine response to wood smoke (CXCL8). We did not show any alteration in macrophage phagocytosis associated with chronic particulate exposure, which is different from reports of acute exposure but consistent with the clinical differences between acute and chronic smoke exposure in human subjects.

A large number of participants underwent bronchoscopy, allowing confidence in the findings. We carefully characterised alveolar macrophage particulate burden as a measure of exposure, and determined *intraphagosomal* function. While *in vitro* challenge models are by their nature different from *in vivo* conditions, and relevant concentrations at cellular interface are debated, our model is consistent with *in vivo* findings: we used respirable fraction particles at concentrations which mimicked cellular appearances of macrophages exposed *in vivo*. Smoke particles were produced from the same firewood sources used by the exposed population, thus providing a relevant exposure model.

### Phagosomal function

We identified a specific defect in phagosomal function associated with natural particulate loading of alveolar macrophages. Despite known crosstalk between oxidative burst and proteolysis pathways, particulate burden was independently associated with reduced oxidative burst, but proteolytic and phagocytic functions were preserved.

These results differ from models of acute exposure which have generally demonstrated decreased phagocytosis [25]. Cellular reactive oxygen species increase following acute exposure, but many methods measure ROS release throughout the cell, including potentially damaging extra-phagosomal production. Compared with our intraphagosomal assay, such methods are more likely to measure cellular oxidative stress than phagocytic killing. In mice, chronic PM_2.5_ exposure causes increased NADPH oxidase activity, by infiltration of bone marrow derived monocytes [26]. In our study, chronic exposures in humans do not have this effect. Particulate toxicity could explain a reduction in oxidative burst, but this does not explain the differential effect on phagocytosis and proteolysis. Other studies report no difference on macrophage viability at the same particulate dose and time interval [27].

Our data provide novel evidence that innate pulmonary immunity is specifically impaired by *in vivo* particulate exposure. Reduced oxidative burst could result in less robust bacterial handling, which is key to innate defence during early pulmonary infection[4]. NADPH oxidase is important in both bacterial killing, and in limiting inflammatory responses by limiting NF-κB activity and the effect of LPS on pro-inflammatory cascades (reduction of NADPH oxidase activity, dampens the pro-inflammatory effect of particulates [28]). These mechanisms are likely to be important in the major observed associations between pulmonary infection and smoke exposure [2].

HAP particulates from biomass fuel combustion contain endotoxin concentrations one hundred times higher than those which cause childhood respiratory disease [11]. In view of the lower oxidative burst in particulate laden macrophages, we hypothesize that chronic inhaled endotoxin down-regulates M1-“activation” phenotype, reducing ROS production analogous to the endotoxin tolerance seen in cigarette smokers [19].

### Release of inflammatory mediators

Wood smoke particles induced AM to produce CXCL8 *in vitro*, which is the prominent systemic cytokine response in wood smoke exposed firefighters [16]. We noted non-significant rises in IL-6 and TNFα which might be explained by our use of lower particulate concentrations than previous studies [10]. Particulate type also alters the effect: WS generates lower level inflammatory responses compared with other particulates in animals [29]. However, we have for the first time used alveolar macrophages obtained from chronically exposed adults who have down-regulated inflammatory AM responses when compared with monocytic cells used in many *in vitro* models [30].

Dampening of LPS cytokine responses has been demonstrated in COPD patients’ mucosa (CXCL8) [31], and alveolar macrophages (TNFα but not CXCL8) [32]. In our study, LPS induced rises in IL-6, CXCL8 and TNFα within bronchoalveolar lavage fluid were reduced by the antioxidant, N-acetylcysteine [33]. We tested the effect of oxidative stress, and showed that CXCL8 was increased in BSO pre-treated cells. The differential in effect (the absence of a measurable effect on IL-6 and TNFα) might be due to relatively larger absolute changes in CXCL8, or the involvement of separate (non-NFΚB) induction pathways. Recent study has shown that redox changes induce CXCL8 in other ways, including Lyn (an Src family kinase), AP-1 binding and histone 3 modifications in the promotor region, [34]

In contrast to CXCL8, CCL2 responses to LPS stimulation were reduced by glutathione depletion. CCL2 is released following acute inflammatory lung injury, reducing AM oxidant production [35]. Under conditions of oxidative stress, reducing CCL2 may therefore prevent excessive inflammation.

Gosset *et al* noted that macrophage glutathione depletion by 90% augments LPS stimulated release of CXCL8 and TNFα [36]. Our BSO treatment effect was less profound (65% reduction), which might explain our observed lack of redox effect. However, Gosset *et al* also demonstrate CXCL8 changes with glutathione reduction of 45%. CXCL8 production is known to be influenced by oxygen metabolites. We are investigating the possibility that our participants’ macrophages had already up-regulated antioxidant defences due to chronic particulate-induced oxidative stress.

## Summary

AM obtained from subjects with prior exposure to high concentrations of respirable particulates show reduced cytokine production at baseline and after further stimulation with particulate generated from wood combustion. Reducing intracellular glutathione increased CXCL8 responses and decreased CCL2 responses in human AM in a similar pattern to that observed in chronically particulate exposed subjects. With LPS stimulation, we did not see an increase in cytokine in BSO treated AM, perhaps due to chronic oxidative stress caused by HAP exposure in this population [36].

In conclusion, particulate exposure *in vivo* is directly associated with impaired macrophage function. It is unlikely that these cellular changes can be specifically altered by antioxidant therapy. These data should, however, provide biological plausibility for public health interventions to reduce HAP with consequent reductions in the burden of pulmonary infection among adults and children living in homes using biomass fuel.

## Supporting Information

S1 Fig Diagram of the gating and analysis strategy used to estimate the intraphagosomal function of alveolar macrophages. (EPS)

S2 Fig Treatment of alveolar macrophages with 0.2mM of BSO for 18 hours did not result in increased toxicity as measured by LDH release into culture medium. Culture medium content of lactate dehydrogenase (LDH) following human alveolar macrophage culture for 18 hours, in the presence BSO 0.2mM or media alone (control). LDH given as proportion of lysed HAM positive control (see Methods). n = 6 in each group.
(EPS)
